# Noninvasive predictors of presence and grade of esophageal varices in viral cirrhotic patients

**DOI:** 10.11604/pamj.2015.20.145.4320

**Published:** 2015-02-17

**Authors:** Lahmidani Nada, El Fakir Samira, Benyachou Bahija, Ibrahimi Adil, Aqodad Nourdine

**Affiliations:** 1 Department of Gastroenterology and Hepatology, University Hospital Hassan II, Faculty of Medicine Fez, Morocco; 2 Department of Epidemiology and Biostatistics, School of Medicine, Fez, Morocco

**Keywords:** Esophageal varices, noninvasive predictors, portal hypertension, cirrhosis

## Abstract

Predicting the presence and the grade of varices by non-invasive methods is likely to predict the need for prophylactic beta blockers or endoscopic variceal ligation. The factors related to the presence of varices are not well-defined. Therefore, the present study has been undertaken to determine the appropriateness of the various factors in predicting the existence and also the grade of esophageal varices. Patients with diagnosis of liver cirrhosis due to hepatitis C or B were included in a retrospective study between January 2001 and January 2010. All the patients underwent detailed clinical evaluation, appropriate investigations, imaging studies (ultrasound with Doppler) and endoscopy at our center. Five variables considered relevant to the presence and grade of varices were tested using univariate and multivariate analysis (logistic regression). Three hundred and seventy two patients with viral liver cirrhosis were included, with 192 (51.6%) males. Platelet count and abundance of ascites were significantly associated with the presence of esophageal varices. However, abundance of ascites, prothrombin time, diameter of the spleen and portal vein were significantly associated with a large varice. In multivariate analysis, platelet count inferior to 100000 was associated with presence of varices (p = 0.04) and only abundance of ascites was associated with large varice. Low Platelet count (< or equal 100000) is associated with the presence of varices in viral cirrhotic patients and abundance of ascites is correlated with the presence of large varices.

## Introduction

Varices are a serious consequence of portal hypertension, and variceal bleeding is a severe complication occurring in up to 30% of patients with cirrhosis. Despite improvement in diagnosis and therapy, mortality from acute variceal bleeding may still reach up to 20%. The most reliable and accurate method to detect the presence of large esophageal varices is an upper gastrointestinal endoscopy. It is now recommended that all patients with established cirrhosis should be screened by upper gastrointestinal endoscopy for the presence of varices at the time of diagnosis. Patients with large varices should be treated with nonselective βeta blockers to reduce the incidence of first variceal bleeding. Predicting the grade of varices by non-invasive methods at the time of diagnosis is likely to predict the need for prophylactic beta blockers or endoscopic variceal ligation in patients with cirrhosis and portal hypertension. The factors related to the presence of varices are not well-defined. Therefore, the present study has been undertaken to determine the appropriateness of the various clinical, biochemical parameters in predicting the existence and also the grade of esophageal varices in viral cirrhotic patients.

## Methods

Patients with diagnosis of liver cirrhosis due to hepatitis C or B were included in a retrospective study between January 2001 and January 2012. All the patients underwent detailed clinical evaluation, appropriate investigations, imaging studies (ultrasound with Doppler) and endoscopy at the department of Gastroenterology and Hepatology. Diagnosis of cirrhosis was based on clinical, biochemical and ultrasonographic finding and /or liver biopsy. History included details and duration of alcoholism, jaundice, ascites, pedal edema and gastrointestinal bleed. Presence or absence of jaundice, ascites, splenomegaly and hepatic encephalopathy was noted. Hemoglobin, platelet count, prothrombin time, blood urea, serum creatinine, liver function tests including serum bilirubin, albumin and transaminases were estimated. At ultrasonogram and Doppler study, the portal vein and spleen diameter along with echo texture of the liver and direction of blood flow were noted. Esophageal varices was assessed in accordance to this classification: Grade I (varices flattening with insufflation), Grade II ( not flattening with insufflation, not confluent, with less than a third of esophageal lumen) and Grade III( large varices not flattening with insufflation and occupying over a third of esophageal lumen or confluent).

We first analyzed clinical, biochemical presentation of patients with Hepatitis B or C virus-related cirrhosis. We performed first a univariate analyses for determining the association of various clinical, laboratories and ultrasonographic variables with the presence or the absence of esophageal varices. P-values below 0.05 were considered significant. All variables that were found to be different between patients with and without Esophageal varices on univariate analysis were included as candidate variables in a forward-conditional step-wise logistic regression analysis to identify independent predictors for the presence of such varices. Ascites was measured in ultrasonography and classified into 2 grades: grade 1 for absent or minimal ascites and grade 2 for mild or important ascites. Previous studies have suggested that a cut off for platelet count around 100000 and 50% for prothrombin time could be used to classify the existence and the grade of esophageal varices.

## Results

From January 2001 to January 2012, 372 patients with viral liver cirrhosis were diagnosed at the department of gastroenterology with a slight male predominance 192 (51.6%). The age ranged from 15 to 96 years with a mean age of 59.4 ±13 years. Patients with bleeding varices represents 42.2% of all cases (n = 157) and 25% of all patients were taking β blockers as primary prevention (Table 1). Clinically, presence of splenomegaly was noted in 87 patients (23%) and 18.8% had encephalopathy. The main Etiology of viral cirrhosis was dominated by hepatitis C (n = 274; 73.6%), in 15 cases there was coinfection B and C. Twenty eight (7.5%) patients had hepatocarcinoma at the time of diagnosis. Ascites was found in 72% of patients by ultrasonography and clinical examination. The mean portal vein diameter of all patients was 14.9 ± 2.8mm (range from 7 to 24 mm). Five variables considered relevant to the presence and grade of varices were tested using univariate analysis. Platelet count (p = 0.0011), prothrombin time (p = 0.04) and abundance of ascites (p = 0.006) were significantly associated with the presence of esophageal varices. However, abundance of ascites, diameter of the spleen and portal vein diameter were significantly associated with size of varices. As shown in Table 2, significant independent association with the presence of esophageal varices was found for the platelet count with a cutoff point of 100000/mm3. Concerning Grade of varices, in multivariate analysis only grade of ascites was associated with large size varices (Table 3).

**Table 1:** Demographic and clinical characteristics of 372 patients

Characteristics	Data
Number of patients	372
Mean age	59.4 + /- 13 years [15-96]
Sex ratio	192/180:H/F:1.06
History of transfusion	22 (5.9%)
Ethylism	32 (8.6%)
Bleeding from esophagel varices	157 (42.2%)
Jaunitis	47 (12.7%)
Collateral veinous circulation	63 (17%)
Splenomegaly	87(23.4%)
Mean of hemoglobin (g/dl)	10.3 + /- 2.8
Mmean of albuminemia (g/l)	29.3 + /-7
High ALAT	167 (44.8%)
Ag HBS positive	83 (22.3%)
VHC positive	274 (73.6%)
VHC + VHB	15 (5.1%)
Splenorenal derivation at ultra sonography	53 (14,2%)
Hepatocarcinoma	28 (7.5%)
Sub cardial varices	49 (13,5%)
Primary prevention by betablockers	95 (25.5%)

**Table 2:** Factors associated with esophageal varices by a logistic regression model

Parameters	OR_a_	95% CI	*p*
Ascites (2 versus1)	0,73	[0,47-1,25]	0,22
Prothrombin time (2versus1)	0,71	[0,45-1,38]	0,23
Platelets count (2 versus1)	1,64	[0,99-2,73]	**0,05**

OR_a_: Adjusted OR ; CI : Confidence Interval

**Table 3:** Factors associated with grade of esophageal varices by a logistic regression model

Parameters	OR_a_	95% CI	*p*
**Ascites (2 versus1)**	1,9	[0,81-2,37]	0,04
**Prothrombin time (2versus1)**	0,61	[0,28-1,22]	0,15
**Platelets count (2versus1)**	0,86	[0,48-1,52]	0,60

OR_a_: Adjusted OR ; CI : Confidence Interval

## Discussion

Up to now, endoscopy has still been the gold standard modality to identifying esophageal varices [1]. However many studies attempt to identify noninvasive factors predicting the presence and the grade of esophageal varices [2, 3]. Several Studies have shown that multiple parameters can be a predictors for the presence of oesophageal varices like splenomegaly, [4–6] ascites [4], spider naevi [7], Child's grade [6, 7], platelet count [7–10], prothrombin time/activity [8], portal vein diameter [11], platelet count/ spleen diameter ratio [11, 12], serum albumin [12], and serum bilirubin [7]. In the present study, on univariate analysis, a platelet count was significantly associated with the presence of esophageal varices in viral related cirrhosis. This factor was also significant on multivariate analysis. Several studies suggest that platelet count may predict the presence of EV in patients with cirrhosis [2, 8, 9]. However, the discriminating threshold for the presence of varices varies widely, ranging between 68,000 and 160,000/mm^3^ [9]. The sensitivities for thrombocytopenia fluctuate from 62% to 100%, and the specificities range from 18% to 77% [11].

Esophageal varice is the direct consequence of spontaneous formation of collateral vessels between portal vein and esophageal veins via left gastric or short gastric veins. Therefore, the presence or absence of EV may reflect the severity of portal hypertension [1, 6]. In a logistic regression study of 143 patients [9], ultrasonographic portal vein diameter greater than 13 mm was one of the independent risk factors for the presence of EV. However, another study [3] failed to confirm the predicting role of portal vein diameter when using a cut-off value of 13 mm in prevalently HCV-related cirrhosis. In our study, in univariate analysis diameter of portal vein was not correlated with the presence of esophageal varices. Amarapurkar et al [13], report that splenomegaly alone was a significant predictor for the development of large esophageal varices. Sharma et al [8] in a prospective study, observed that splenomegaly and platelet count were the independent predictors for the presence of large varices. From the present study, only importance of ascites was correlated with the presence of large varices.

## Conclusion

Our data suggest that easily obtainable non-invasive markers are effective for predicting esophageal varices in cirrhosis related to hepatitis C and/or B. These predictors may help physicians to initiate appropriate primary pharmacological prophylaxis in areas where endoscopy is not easily accessible. In areas where endoscopy is accessible, a non-invasive predictor, as in this study, can help physicians to initiate drug therapy while waiting for the endoscopy procedure specially in bleeding situation.
